# Effectiveness of hallux valgus surgery on patient quality of life: a systematic review and meta-analysis

**DOI:** 10.1080/17453674.2020.1764193

**Published:** 2020-05-14

**Authors:** Luis Enrique Hernández-Castillejo, Vicente Martínez Vizcaíno, Miriam Garrido-Miguel, Iván Cavero-Redondo, Diana P Pozuelo-Carrascosa, Celia Álvarez-Bueno

**Affiliations:** a Health and Social Research Center, Universidad de Castilla La Mancha, Cuenca, Spain;; b Facultad de Ciencias de la Salud, Universidad Autónoma de Chile, Talca, Chile;; c Universidad Politécnica y Artística del Paraguay, Asunción, Paraguay

## Abstract

Background and purpose — The quality of life (QoL) of patients with hallux valgus (HV) usually improves postoperatively. Evidence regarding the effect of HV surgery on different domains of patient QoL remains inconclusive. This systematic review and meta-analysis estimates the effect of HV surgery on patient QoL through distinguishing effects on physical domains (comprising physical function and body pain domains) using the EuroQol-5D, short form (SF) health survey-12, and SF-36 QoL scales and a visual analogue scale (VAS) score and mental and social domains using QoL scales.

Patients and methods — MEDLINE, EMBASE, Cochrane Library, and Web of Science databases were systematically searched from inception to March 2019 for studies on the effect of HV surgery on patient QoL. A standardized mean difference score was calculated for each specific QoL domain (mental, social, pain, physical, and VAS) using Cohen’s d index. The pooled effect size (ES) was estimated using a random-effects model based on the DerSimonian and Laird method.

Results — From 12 published studies selected, the estimated pooled ES for QoL was 1.01 (95% confidence interval [CI] 0.52–1.51; I^2^ = 87%) for body pain and 0.43 (CI 0.31–0.55, I^2^ = 35%) for physical function. Regarding the composite mental and social domains of QoL, the pooled ES estimates were 0.24 (CI 0.00–0.47, I^2^ = 80%) and 0.42 (CI 0.21–0.63, I^2^ = 6.4%), respectively. The pooled difference in means for the VAS score was –4.1 (CI –4.5 to –3.6, I^2^ = 90%).

Interpretation — Our data showed that HV surgery decreased patients’ perceptions regarding pain. Furthermore, the data confirmed that HV surgery increased patients’ QoL, particularly concerning physical and social domains.

More than 200 different surgical procedures have been developed for treating hallux valgus (HV) (Myerson 2000, Magnan et al. 2005, Easley and Trnka 2007). Evidence supporting these differing surgical approaches for HV remains inconclusive; therefore, patient-reported outcome measures (PROMs) could be decisive for favoring one approach over another among these numerous surgical alternatives. PROMs are typically classified as pain scales, general scales, and region-specific outcomes. For region-specific PROMs, the Manchester-Oxford Foot Questionnaire (MOXFQ), the Foot and Ankle Outcome Score (FAOS), the Self-reported Foot and Ankle Score (SEFAS) (Schrier et al. 2015), and the American Orthopaedic Foot and Ankle Society (AOFAS) score (Hunt and Hurwit 2013, Arbab et al. 2019, Nilsdotter et al. 2019) have been developed and validated. Generic PROMs, including scales such as the EuroQol-5D (EQ-5D), the Short Form-12 Health Survey (SF-12), and the SF-36, have also been used.

While surgical treatment for HV is more effective than nonoperative or no treatment (Klugarova et al. 2017), there are no substantial differences among surgical alternatives in terms of pain or other region-specific outcomes such as recurrence rates or nerve injury. However, there are few studies reporting results on HV surgery in terms of quality of life (QoL), and the effectiveness of HV surgery on different domains in terms of patient QoL remains inconclusive.

This systematic review and meta-analysis aimed to estimate the effect of HV surgery on patient QoL through distinguishing physical domains (including physical function and body pain domains) using the QoL scales EQ-5D, SF-12, and SF-36, and visual analogue scale (VAS) scores, and mental and social domains using QoL scales.

## Patients and methods

This systematic review and meta-analysis was undertaken following the Preferred Reporting Items for Systematic Reviews and Meta-Analyses (Moher et al. 2010), Statement, and guided by the Cochrane Collaboration Handbook (Higgins and Green 2008).

### Search strategy

The electronic databases MEDLINE (via PubMed), EMBASE, Cochrane Library, and Web of Science were systematically searched to identify relevant publications. The systematic search included the following keywords: “hallux valgus”, foot, ankle, metatarsal, surgery, osteotomy, “quality of life”, SF-12, FAOS, SF-36, SEFAS, VAS, EQ-5D, MOXFQ, AOFAS, LLFI, NRS, and SAFE-Q. The search was conducted from database inception to March 2019. The complete search strategy for MEDLINE is reported in Table 1 (see Supplementary data).

**Table 2.** Characteristics of the studies included in this systematic review and meta-analysis

	Sample size		Mean		Preoperative	Postoperative	Follow up (months)
Reference (country)	women	men	age	Scales	mean (95% CI or SD)	mean (95% CI or SD)	
Al Nammari et al. 2015 (UK)	43	4	56	MOXFQ	MOXFQ: 74 (30–100)	MOXFQ: 13 (0–61)	14–60
					• Walking: 75 (29–100)	• Walking: 12 (0–66)	
					• Pain: 79 (35–100)	• Pain: 16 (0–69)	
					• Social: 67 (25–100)	• Social: 11 (0-69)	
Chen et al. 2016 (Singapore)							
Mild residual pain	65	5	52	VAS	• VAS: 5 (4–7)	• VAS: 0 (0–4)	6–24
				PCS	• PCS: 50 (39–54)	• PCS: 51 (43–54)	
				MCS	• MCS: 53 (46–60)	• MCS: 56 (49–60)	
Several residual pain	19	1			• VAS: 6 (4–8)	• VAS: 0 (0–3)	
					• PCS: 45 (35–52)	• PCS: 53 (46–59)	
					• MCS: 49 (42–61)	• MCS: 48 (42–52)	
Chen et al. 2015 (Singapore)							
Control	375	28	51	VAS	• VAS: 5 (4–5)	• VAS: 1 (0–1)	24
				PCS	• PCS: 55 (53–57)	• PCS: 85 (84–87)	
				MCS	• MCS: 55 (53–56)	• MCS: 55(54–56)	
Obese	44	5	55		• VAS: 5 (5–6)	• VAS: 1 (0–2)	
					• PCS: 49 (44–54)	• PCS: 84 (79–88)	
					• MCS: 53 (50–56)	• MCS: 54 (51–57)	
Choi et al. 2013 (USA)	48	3	59	VAS	VAS: 5.8 (1.9)	VAS: 1.1 (1.4)	
				SF-36	SF-36:	SF-36:	
					• Physical: 46 (8.9)	• Physical: 52 (7.3)	12–24
					• Mental: 55 (6.8)	• Mental: 55 (6.9)	
Dawson et al. 2006 (UK)	95	5	50	MOXFQ	MOXFQ:	MOXFQ:	12
				SF-36	• Foot pain: 53 (SD 21)	• Foot pain: 20 (SD 21)	
					• Walking: 45 (SD 25)	• Walking: 16 (SD 23)	
					• Social: 47 (SD 23)	• Social: 12 (SD 19)	
					SF 36:	SF 36:	
					• Pain: 62 (SD 24)	• Pain: 77 (SD 21)	
					• Physical: 75 (SD 23)	• Physical: 85 (SD 19)	
					• Role P: 75 (SD 27)	• Role P: 86 (SD 25)	
					• Mental: 71 (SD 17)	• Mental: 78 (SD 16)	
					• Role M: 83 (SD 23)	• Role M: 91 (SD 18)	
					• Vitality: 57 (SD 20)	• Vitality: 63 (SD 18)	
					• Social: 78 (SD 23)	• Social: 85 (SD 21)	
					• Health: 76 (SD 19)	• Health: 80 (SD 17)	
Hogea et al. 2017 (Romanian)	35	21	44.4	VAS	VAS: 59 (SD 31)	VAS: 20 (SD 23)	24–60
				EQ5-D	EQ5-D:	EQ5-D:	
					• Anxiety: 1.9 (SD 0.65)	• Anxiety: 1.0 (SD 0.40)	
					• Usual activities: 2.9 (SD 0.63)	• Usual activities: 2.9 (SD 0.55)	
					• Self-care: 1.9 (SD 0.82)	• Self-care: 1.9 (SD 0.62)	
					• Mobility: 2.6 (SD 0.33)	• Mobility: 1.5 (SD 0.32)	
					• Pain: 2.9 (SD 0.94)	• Pain: 1.7 (SD 0.65)	
Kaufmann et al. 2018 (Austria)							
Open	19	3	44	VAS	VAS: 6	VAS: 0	1.5–9
Percutaneous	21	4	52		VAS: 5	VAS: 1	
Lai et al. 2017 (Singapore)							
Open	52	6	54	VAS	VAS: 4.9 (SD 2.6)	VAS: 0.4 (SD 1.5)	6–24
				SF-36	SF-36:	SF-36:	
					• Physical: 82 (SD 19)	• Physical: 83 (SD 20)	
					• Mental: 86 (SD 15)	• Mental: 86 (SD 15)	
Percutaneous	25	4			VAS: 4.0 (SD 2.9)	VAS: 0.7 (SD 1.9)	
					SF-36:	SF-36:	
					• Physical: 76 (SD 22)	• Physical: 83 (SD 22)	
					• Mental: 79 (SD 18)	• Mental: 85 (SD 15)	
Lee et al. 2017 (Australia)							
Open	22	3	53.4	VAS	VAS: 6.9 (SD 1.7)	VAS: 0.5 (SD 1.1)	6
Percutaneous	23	2	52.6		VAS: 7.1 (SD 1.5)	VAS: 0.3 (SD 0.9)	
Milczarek et al. 2017 (Poland)							
Normal BMI	71	(W + M)	52	VAS	VAS: 5 (4–6)	VAS: 2 (1–3)	24
High BMI	62	(W + M)	61		VAS: 5 (4–6)	VAS: 2 (1–2)	
Niki et al. 2017 (Japa)n	92	8	62	SF-36	SF-36:	SF-36:	9–12
				SAFE-Q	• Pain: 53 (SD 23)	• Pain: 74 (SD 21)	
					• Physical: 70 (SD 23)	• Physical: 80 (SD 20)	
					• Role P: 70 (SD 28)	• Role P: 85 (SD 18)	
					• Mental: 67 (SD 20)	• Mental: 71 (SD 19)	
					• Role M: 67(SD 20)	• Role M: 87 (SD 18)	
					• Vitality: 56 (SD 19)	• Vitality: 64 (SD 17)	
					• Social: 76 (SD 25)	• Social: 85 (SD 21)	
					• Health: 57 (SD 20)	• Health: 61 (SD 18)	
					SAFE-Q:	SAFE-Q:	
					• Pain: 59 (SD 24)	• Pain: 87 (SD 13)	
					• Physical: 71 (SD 25)	• Physical: 87 (SD 16)	
					• Social: 68 (SD 30)	• Social: 90 (SD 16)	
					• Shoe-related: 37 (SD 20)	• Shoe-related: 71 (SD 23)	
					• General health: 64 (SD 27)	• General health: 90 (SD 15)	
Saro et al. 2007 (Sweden)	94	6	48	SF-36	SF-36:	SF-36:	12
					• Pain: 58 (SD 22)	• Pain: 75 (SD 24)	
					• Physical: 83 (SD 17)	• Physical: 86 (SD 19)	
					• Role P: 77 (SD 37)	• Role P: 86 (SD 31)	
					• Mental: 77 (SD 18)	• Mental: 85 (SD 16)	
					• Role M: 81 (SD 34)	• Role M: 91 (SD 26)	
					• Vitality: 62 (SD 23)	• Vitality: 71 (SD 22)	
					• Social: 83 (22)	• Social: 89 (SD 21)	
					• Health: 77 (SD 20)	• Health: 85 (SD 16)	

Abbreviations: W: Women; M: Men, VAS: visual analogical scale, SF: Short Form-36 Health Survey, MOXFQ: Manchester-Oxford Foot Questionnaire, PCS: Physical Component Score, MCS: Mental Component, Score, SD: Standard deviation. EQ5-D: EuroQol-5D, BMI: Body-mass index, SAFE-Q: Self-administered foot evaluation Questionnaire

### Eligibility

Studies on the effect of HV surgery on patient QoL measured through QoL scales other than the AOFAS were included. The inclusion criteria were as follows: (i) patients > 16 years old; (ii) open (i.e., Chevron, Scarf, Kramer, or Bösch) or minimally invasive surgery procedures; (iii) QoL reports on physical (including physical function and body pain domains of QoL scales, and a VAS score), mental and social domains (including social function, emotional role, mental health, vitality, and general health) using QoL scales other than the AOFAS; (iv) randomized controlled trials (RCTs), non-randomized experimental studies, and single-arm pre–post studies, and; (v) reports written in English or Spanish.

We excluded studies reporting data on foot or ankle pathologies other than HV, as well as patients who had undergone HV revision surgery.

### Data extraction and risk of bias assessment

The following data were extracted from the included studies: (1) author, (2) year of publication, (3) country of study, (4) number of participants according to sex (in the control and intervention groups), (5) mean age, (6) QoL scale and domains reported, (7) type of intervention, and; (8) end-point measures (in months).

After concealing information regarding authors, affiliations, date, and source of each manuscript, 2 investigators (CA-B and LH-C) independently assessed the risk of bias of included studies, and inconsistencies were resolved through consensus or through consulting a third researcher (VM-V).

The Quality Assessment Tool for Observational Cohort and Cross-sectional Studies from the National Heart, Lung, and Blood Institute (NIH) (National Heart, Lung, and Blood Institute, 2019) was used to assess the risk of bias of the pre–post studies. This tool evaluates 7 domains including selection bias, study design, confounders, blinding, data collection method, withdrawals, and dropouts. Each domain could be considered as strong, moderate, or weak (Table 3, Supplementary data).

The Cochrane Risk of Bias tool for randomized trials (RoB 2) (a revised tool to assess risk of bias in randomized trials) was used to assess the risk of bias in the RCTs. This tool evaluates 6 domains: randomization, assignment to intervention, adherence to intervention, missing outcome data, measurement of the outcome, and selection of the reported results. Each domain could be considered to have low bias concerns, some bias concerns, or a high risk of bias (Table 4, Supplementary data).

### Statistics

A standardized mean difference score was calculated for each specific QoL reported, using Cohen’s d index (Cohen 1977), in which positive ES values indicated higher outcome scores in favor of the intervention group. Cohen’s d values were interpreted as follows: 0.2 (considered a weak effect), 0.5 (considered a moderate effect), 0.8 (considered a strong effect), and > 1.0 (considered a very strong effect). Pooled ES estimates for pre–post interventions and their 95% confidence intervals (CIs) were estimated using a random-effects model based on the DerSimonian and Laird method (DerSimonian and Kacker 2007) through distinguishing physical domains (including body pain and physical function) using QoL scales, and mental and social domains. For the VAS score, the pooled difference in means for pre–post interventions and their CIs were calculated, in which negative values indicated better VAS scores in favor of the intervention group. Heterogeneity across studies was assessed using the I^2^ statistic (Higgins and Thompson 2002), with values of < 30%, ≤ 30% to < 60%, ≤ 60% to 85%, and > 85% considered as indicating not important, moderate, substantial, and considerable heterogeneity, respectively (Higgins and Green 2008).

Some considerations should be noted. First, only studies providing complete data for pre- and post-intervention measurements were included in the meta-analysis. Second, when studies provided 2 measures for the same domain, both measures were pooled to calculate the mean ES. Third, data from different cohorts were considered as independent samples. Finally, when studies reported data for several follow-up points, only the data in relation to the longest follow-up were considered.

The influence of each study in the pooled ES estimates was examined using sensitivity analyses. Additionally, meta-regression analyses were conducted to assess the influence of mean age and the percentage of females on the magnitude of the effect of HV surgery on QoL domains. Egger’s regression asymmetry test (Sterne et al. 2001, Tanner-Smith et al. 2019) was used to assess publication bias. The significance value of the pooled ES was estimated based on a 95% CI. Statistical analyses were performed using STATA SE software, version 15 (StataCorp, College Station, TX, USA).

### Ethics, funding, and potential conflicts of interest

The protocol for this systematic review and meta-analysis was registered on PROSPERO (Registration number: CRD42019121120). There is no funding. There are no potential conflicts of interest.

## Results

### Systematic review

A literature search retrieved 3,924 studies and, after exclusion of non-relevant studies, 47 full-text studies were assessed for eligibility. Of these, 12 studies were included in this systematic review and meta-analysis (Figure 1, Supplementary data).

Table 2 presents data from the included studies, which had been published between 2006 and 2018, and included 1 RCT and 10 single-arm pre–post studies. These studies had been conducted in 9 countries (the United Kingdom, Singapore, the United States, Romania, Austria, Australia, Poland, Japan, and Sweden), and comprised 1,313 patients who had undergone HV surgery, and whose ages ranged from 44 to 62 years. End-point measurements were obtained from 4 weeks to 5 years.

The evaluation of the clinical outcomes was performed using the following tools: MOXFQ (Dawson et al. 2006, Al-Nammari et al. 2015), VAS (Dawson et al. 2006, Choi et al. 2013, Chen et al. 2015, 2016, Hogea et al. 2017, Lee et al. 2017, Milczarek et al. 2017, Lai et al. 2018, Kaufmann et al. 2019), the SF-36 (Saro et al. 2007, Choi et al. 2013), EuroQol-5D (EQ5-D) (Kaufmann et al. 2019), and the SAFE-Q (Niki et al. 2017).

### Risk of bias

According to the Quality Assessment Tool for Observational Cohort and Cross-sectional Studies, all pre–post studies included in the meta-analysis were considered as having a moderate risk of bias. When individual domains using the tool were assessed, all the studies included information relating to the representativeness of the sample and the description of the intervention; however, all the studies were limited in terms of sample eligibility and blinding (Table 3, Supplementary data).

The study, analyzed using a RoB 2.0 tool (Cochrane Collaboration n.d.) for randomized trials, was categorized as having a high risk of bias (Table 4, Supplementary data).

### Meta-analysis

**Figure 2.**
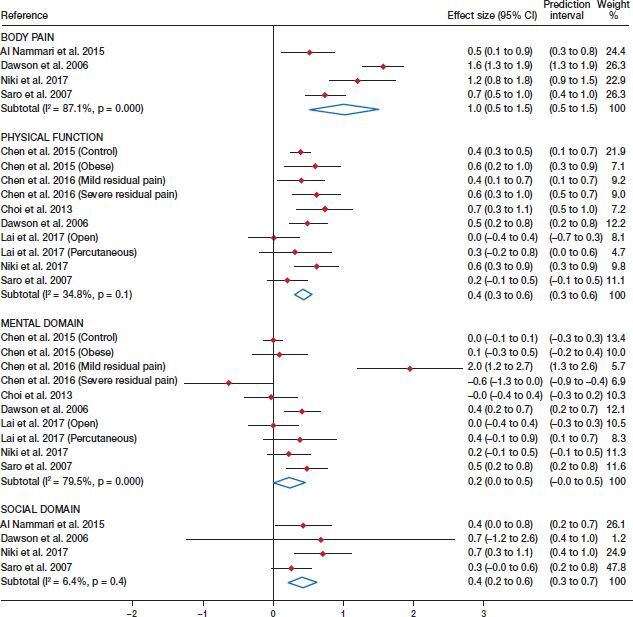
Forest plot for ES of the body pain and physical function components, and mental and social domains.

The pooled ES estimates for the QoL physical domain scores were 1.01 (CI 0.52–1.51; I^2^ = 87%) for body pain and 0.43 (CI 0.31–0.55, I^2^ = 35%) for physical function (Figure 2).

Concerning the mental and social domains of QoL, the pooled ES estimates were 0.24 (CI 0.00–0.47, I^2^ = 80%) and 0.42 (CI 0.21–0.63, I^2^ = 6.4%), respectively (Figure 2).

**Figure 3.**
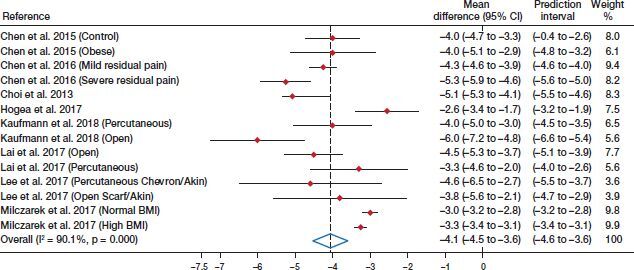
Forest plot for the difference in means of the visual analogue scale (VAS).

Finally, the pooled difference in means for the VAS score was –4.1 (CI –4.5 to –3.6, I^2^ = 90%) (Figure 3).

### Sensitivity analysis and meta-regressions

When the effect of individual studies was examined by removing studies from the analysis one at a time, only the estimate for the mental domain of QoL was modified after removing the samples from the Chen et al. (2016; severe residual pain) and Choi et al. studies (2013; Table 5, Supplementary data).

Additionally, meta-regressions showed that only the percentage of females affected the association between HV surgery and the VAS score (Table 6), Supplementary data.

### Publication bias

Evidence of publication bias was identified using funnel plot asymmetry and Egger’s test for the effect of HV surgery on the VAS score (p = 0.02) (Figure 4, Supplementary data).

## Discussion

This systematic review and meta-analysis aimed to estimate the effect of HV surgery on patient QoL through distinguishing the effects on the physical domain and VAS scores, and mental and social domains, using QoL scales. Our findings showed that HV surgery resulted in decreased body pain and improved physical function, and improved the social domain of patient QoL, although it did not modify the mental QoL domain score.

Most QoL scales include patients’ perceptions of different domains of physical health status. Although the validity and reliability of the VAS and QoL scales used in the included studies have been confirmed (Schrier et al. 2015), the numerous surgical approaches and the small number of studies meeting the inclusion criteria could have affected our results. Furthermore, because of the scarcity of studies, we could not undertake stratified analyses involving differing surgical techniques to examine subsequent differences in the effects on QoL and pain. However, our findings show that HV surgery had a positive effect on the physical domain, regardless of the scale used to measure it.

Anxiety and depression are common disorders that, similar to HV, have been reported to be more prevalent in women (Shakked et al. 2018). Moreover, because those with anxiety and/or depression have a greater percentage of severe deformities than those unaffected by these mental disorders, they score lower in the mental domain of QoL at baseline (Cody et al. 2018, Shakked et al. 2018). Our findings, which are in line with previous studies (Dawson et al. 2006, Chen et al. 2016), suggest no improvement after HV surgery in the mental domain of QoL. Because most included studies did not report the percentage of participants with a diagnosis of these mental disorders, it is not possible to know whether anxiety and/or depressive disorders explain this lack of improvement in the mental health domain of QoL following HV surgery. It is important that HV surgery be approached in a multidisciplinary and multidimensional manner (Hogea et al. 2017), especially for patients with comorbidities and high risks concerning mental health, such as depression, or general health issues.

Our assessment of multiple domains of QoL scales aimed to objectively measure the effectiveness of HV surgery on patient daily life. Each domain included in these scales (physical, mental, and social) is related to QoL and these are closely related to each other. To ensure an appropriate patient evaluation and follow-up after HV surgery, besides the AOFAS questionnaire commonly used by orthopedic surgeons, the use of different QoL scales is encouraged (Thordarson et al. 2005, Fraissler et al. 2016). It is not advisable to use a single instrument to collect quality orthopedic data as selection is dependent on the population being examined and the questions being asked, and all PROMs should be appropriately referenced in the studies. We suggest that for patient-reported evaluation of HV surgery, one of the region-specific validated PROMs (such as the MOXFQ, FAOS, and SEFAS) would be a good option and should be used in combination with a generic PROMs tool measuring QoL (such as EQ-5D, SF-12, and SF-36) (Schrier et al. 2015, Kitaoka et al. 2018, Arbab et al. 2019, Nilsdotter et al. 2019).

A recent systematic review (Barg et al. 2018), which reviewed 229 articles that included 16,237 surgeries, found unfavorable outcomes of surgical treatment for HV deformity and highlighted the limited quality of the evidence provided by the published studies involving predominantly retrospective case series, and the lack of sufficient studies to conduct analyses according to type of surgery. Moreover, although less-invasive procedures for HV have generated great enthusiasm, another systematic review (Bia et al. 2018), which aimed to synthesize the clinical evidence for percutaneous HV surgery, reported inconclusive results.

Our meta-regression analyses using mean age and percentage of females showed no changes in the pooled ES. Previous findings in this regard have been inconclusive (Chou et al. 2008). While some authors state that age is not a significant predictor for pain scores (Chou et al. 2008, Fernández et al. 2017), others state that age is related to some subscale scores (Niki et al. 2013). Furthermore, although the prevalence of HV is higher among females, the predictive effect of HV surgery does not appear to depend on patients’ sex, age, or severity of HV (Chou et al. 2008, Fraissler et al. 2016).

Our study had some limitations. First, the study had limitations common to systematic reviews and meta-analyses such as selection bias and limited availability of complete information from study reports. Second, although there was no evidence of publication bias from Egger’s test in most of the domains analyzed, results from unpublished studies could have modified the results of our meta-analysis. Third, several factors could have influenced both clinician- and patient-related outcomes, such as the type of surgical approach, surgeons’ skills, and comorbidities (Cöster et al. 2014), and these were not controlled for in the analyses owing to the scarcity of information in the included studies. Fourth, we were unable to establish cause–effect inferences due to the nature of the observational studies selected. Finally, language selection bias could not be ruled out since studies published only in English and Spanish were included.

In conclusion, this systematic review and meta-analysis provided a synthesis of the evidence that HV surgery decreased patient perception of pain. Furthermore, our data showed that HV surgery increased patient QoL, especially in the physical and social domains. This study highlights the need to include PROM measures, such as pain or QoL, through region-specific validated scales prior to and post-HV surgery.

## Supplementary Material

Supplemental Material

## Supplementary data

Tables 1 and 3–6 and Figures 1 and 4 are available as supplementary data in the online version of this article, http://dx.doi.org/10.1080/17453674.2020.1764193
