# Supplementary material for: Effectiveness of hallux valgus surgery on patient quality of life: a systematic review and meta-analysis
Source: Acta Orthop. 2020 May 14;91(4):450–6. doi: 10.1080/17453674.2020.1764193 (PMC8023907; doi:10.1080/17453674.2020.1764193)
Supplement: Supplemental Material [file IORT_A_1764193_SM0948.pdf]

Supplementary data

Table 1. Search strategy for Medline

"hallux valgus" OR "foot" OR "ankle" OR "metatarsal"
AND
"surgery" OR "osteotomy"
AND
quality of life OR SF-12 OR FAOS OR SF-36 OR SEFAS OR VAS OR EQ-5D OR MOXFQ OR AOFAS OR LLFI OR NRS OR SAFE-Q

Table 3. Risk of bias assessment for pre-post studies using the 'Quality Assessment Tool for Before–After (Pre-Post) Studies with No Control Group'

Reference	1	2	3	4	5	6	7	8	9	10	11	12	Quality rating [a]
Al Nammari et al. 2015	Yes	No	Yes	CD	NR	Yes	Yes	No	NR	No	NA	NA	Fair
Chen et al. 2016	Yes	No	Yes	No	Yes	Yes	Yes	No	NR	Yes	NA	NA	Fair
Chen et al. 2015	Yes	Yes	Yes	No	Yes	Yes	Yes	No	NR	Yes	NA	NA	Fair
Choi et al. 2013	Yes	Yes	Yes	No	NR	Yes	Yes	No	NR	No	NA	NA	Fair
Dawson et al. 2006	Yes	Yes	Yes	No	Yes	Yes	Yes	No	NR	Yes	NA	NA	Fair
Hogea et al. 2017	Yes	No	Yes	No	No	Yes	Yes	No	NR	No	NA	NA	Fair
Lai et al. 2017	Yes	No	Yes	No	Yes	Yes	Yes	No	NR	Yes	NA	NA	Fair
Lee et al. 2017	Yes	Yes	Yes	No	No	Yes	Yes	No	NR	Yes	NA	NA	Fair
Milczarek et al. 2017	Yes	Yes	Yes	No	No	Yes	Yes	No	NR	Yes	NA	NA	Fair
Niki et al. 2017	Yes	Yes	Yes	No	No	Yes	Yes	No	NR	Yes	Yes	NA	Fair
Saro et al. 2007	Yes	Yes	Yes	No	No	Yes	Yes	No	NR	Yes	NA	NA	Fair

[a] Risk of bias rating (Low (75–100%), Moderate (25–75%), or High (0–25%)). Good, Fair or Poor.
Abbreviations: CD = cannot determine, NA = not applicable, NR = not reported.

1. Objective clearly stated;
2. Eligibility criteria described;
3. Representative patient population;
4. All eligible participants enrolled in study;
5. Sufficient sample size;
6. Intervention described;
7. Outcome measures specified;
8. Outcome assessors blinded;
9. Loss to follow-up;
10. Statistical analysis of outcome measures before and after intervention;
11. Interrupted time-series design;
12. Individual data used for group-level effects.

Table 4. Risk of bias assessment of randomized controlled trial using the 'RoB 2.0 tool for randomized trial'

Reference	Randomization	Interventions	Missing outcome data	Outcome measurement	Reported results	Overall
Kauffmann et al. 2018	Low	Some concerns	Low	High	Low	High

Table 5. Sensitivity analysis by removing one by one of the studies

Reference	Effect size (95% CI)
BODY PAIN	
Al Nammari et al. 2015	1.17 (0.62 to 1.72)
Dawson et al. 2006	0.79 (0.46 to 1.13)
Niki et al. 2017	0.95 (0.32 to 1.59)
Saro et al. 2007	1.10 (0.45 to 1.75)
PHYSICAL FUNCTION	
Chen et al. 2015 (Control)	0.44 (0.29 to 0.59)
Chen et al. 2015 (Obese)	0.42 (0.29 to 0.55)
Chen et al. 2016 (Mild residual pain)	0.43 (0.30 to 0.57)
Chen et al. 2016 (Severe residual pain)	0.41 (0.28 to 0.54)
Choi et al. 2013	0.41 (0.29 to 0.52)
Dawson et al. 2006	0.42 (0.29 to 0.56)
Lai et al. 2017 (Open)	0.45 (0.35 to 0.55)
Lai et al. 2017 (Percutaneous)	0.44 (0.31 to 0.57)
Niki et al. 2017	0.41 (0.28 to 0.54)
Saro et al. 2007	0.46 (0.33 to 0.58)
MENTAL DOMAIN	
Chen et al. 2015 (Control)	0.27 (0.00 to 0.55)
Chen et al. 2015 (Obese)	0.27 (0.00 to 0.51)
Chen et al. 2016 (Mild residual pain)	0.14 (–0.03 to 0.32)
Chen et al. 2016 (Severe residual pain)	0.29 (0.06 to 0.53)
Choi et al. 2013	0.27 (0.01 to 0.52)
Dawson et al. 2006	0.21 (–0.04 to 0.47)
Lai et al. 2017 (Open)	0.27 (0.00 to 0.52)
Lai et al. 2017 (Percutaneous)	0.22 (–0.02 to 0.47)
Niki et al. 2017	0.24 (–0.02 to 0.50)
Saro et al. 2007	0.20 (–0.05 to 0.45)
SOCIAL DOMAIN	
Al Nammari et al 2015	0.46 (0.10 to 0.81)
Dawson et al. 2006	0.43 (0.17 to 0.69)
Niki et al. 2017	0.32 (0.09 to 0.55)
Saro et al. 2007	0.57 (0.20 to 0.85)
VAS SCORE	
Chen et al. 2015 (Control)	–4.1 (–4.5 to –3.6)
Chen et al. 2015 (Obese)	–4.1 (–4.5 to –3.6)
Chen et al. 2016 (Mild residual pain)	–4.0 (–4.5 to –3.6)
Chen et al. 2016 (Severe residual pain)	–3.9 (–4.4 to –3.5)
Choi et al. 2013	–4.0 (–4.4 to –3.5)
Hogea et al. 2017	–4.2 (–4.7 to –3.7)
Kaufmann et al. 2018 (Percutaneous)	–4.1 (–4.5 to –3.6)
Kaufmann et al. 2018 (Open)	–3.9 (–4.4 to –3.5)
Lai et al. 2017 (Open)	–4.0 (–4.5 to –3.6)
Lai et al. 2017 (Percutaneous)	–4.1 (–4.6 to –3.6)
Lee et al.2017 (Percutaneous Akin/Chevron)	–4.0 (–4.5 to –3.6)
Lee et al.2017 (Open Scarf/Akin)	–4.1 (–4.5 to –3.6)
Milczarek et al. 2017 (Normal BMI)	–4.2 (–4.7 to –3.7)
Milczarek et al. 2017 (High BMI)	–4.2 (–4.8 to –3.6)

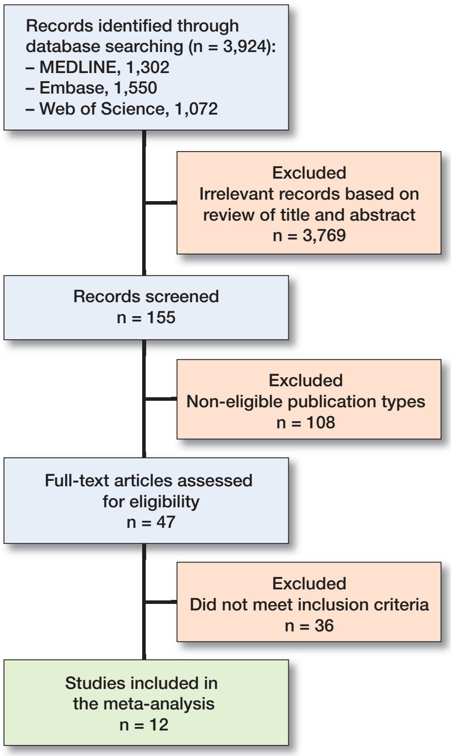

Figure 1. PRISMA flow chart

Table 6. Meta-regression of the hallux valgus surgery outcomes by participants' mean age and percentage of women of included studies

Outcome	I^2	Mean age ß (95% CI))	p-value	I^2	% women ß (95% CI)	p-value
Body pain	91.1	–0.00 (–0.06 to 0.05)	0.7	87.6	0.15 (–0.46 to 0.75)	0.4
Physical function	20.4	0.02 (–0.01 to 0.06)	0.1	35.8	0.02 (–0.03 to 0.80)	0.3
Mental domain	81.4	–0.02 (–0.13 to 0.08)	0.6	81.7	–0.01 (–0.15 to 0.14)	0.9
Social domain	48.5	0.02 (–0.07 to 0.11)	0.4	66.1	–0.00 (–0.36 to 0.36)	1.0
VAS score	90.6	0.01 (–0.12 to 0.14)	0.9	52.0	–0.04 (–0.07 to –0.01)	0.01

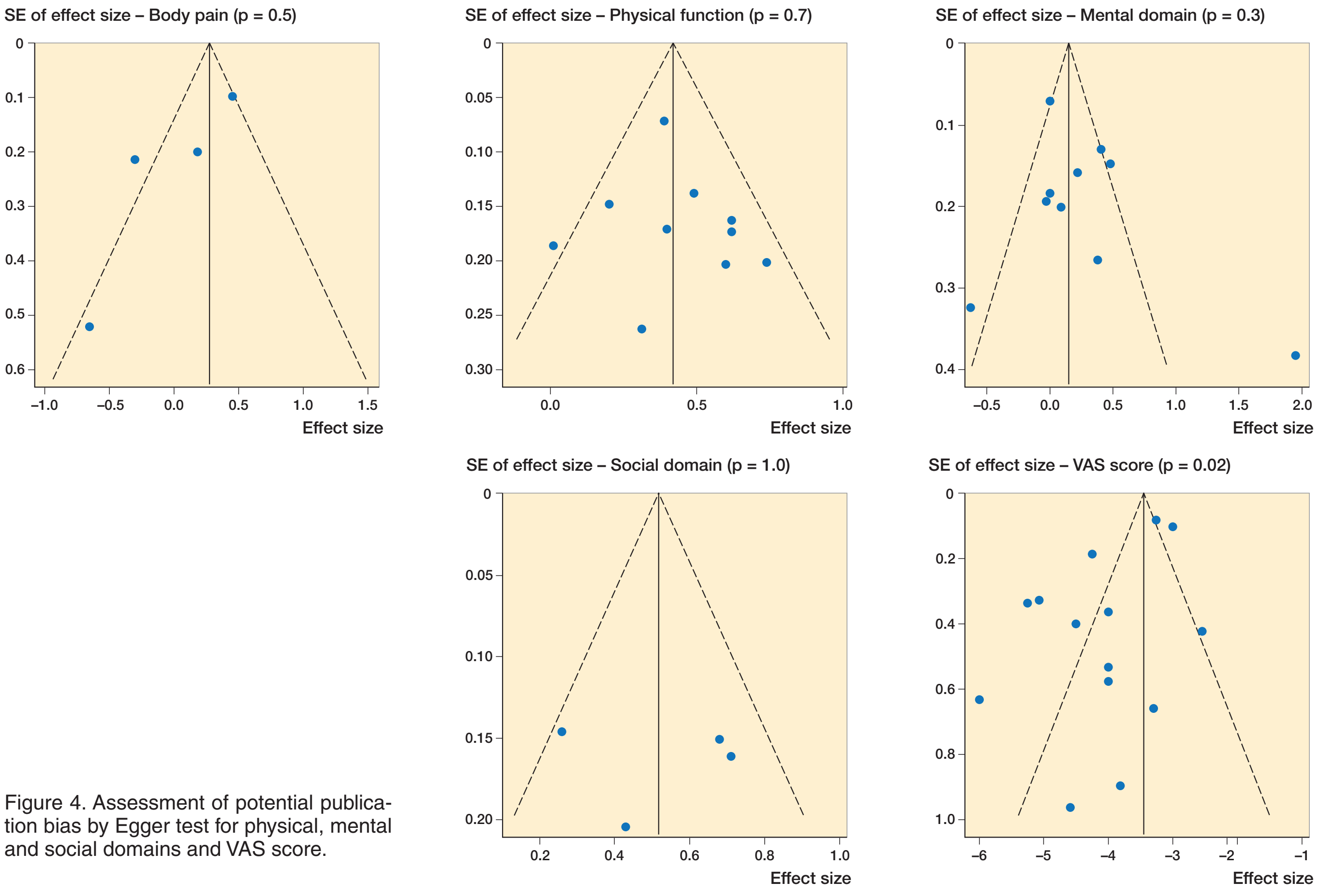

Figure 4. Assessment of potential publication bias by Egger test for physical, mental and social domains and VAS score.
